# RETRACTED: Bouam et al. Rapid Isothermal Amplification for the Buccal Detection SARS-CoV-2 in the Context of Out-Patient COVID-19 Screening. *J. Clin. Med.* 2021, *10*, 2643

**DOI:** 10.3390/jcm14082569

**Published:** 2025-04-09

**Authors:** Amar Bouam, Jean-Jacques Vincent, Elisabeth Le Glass, Lionel Almeras, Pierre-Yves Levy, Hervé Tissot-Dupont, Jean-Christophe Lagier, Pierre-Edward Fournier, Didier Raoult, Michel Drancourt

**Affiliations:** 1 Aix-Marseille-Université, IRD, MEPHI, IHU Méditerranée Infection, 13005 Marseille, France; 2 POCRAMé, 13005 Marseille, France; 3 IHU Méditerranée Infection, 13005 Marseille, France; 4 Aix-Marseille-Université, IRD, VITROME, IHU Méditerranée Infection, 13005 Marseille, France; 5 Unité Parasitologie et Entomologie, Département Microbiologie et Maladies Infectieuses, Institut de Recherche Biomédicale des Armées, 13005 Marseille, France

The *Journal of Clinical Medicine* Editorial Office retracts the article, “Rapid Isothermal Amplification for the Buccal Detection SARS-CoV-2 in the Context of Out-Patient COVID-19 Screening” [1], cited above.

Following publication, concerns were brought to the attention of the Editorial Office regarding the extent to which this article [1] fully adheres to national regulations governing research involving human participants.

Adhering to our complaints procedure, an investigation was conducted by the Editorial Office and Editorial Board that confirmed that the authors failed to apply and receive approval from the Comité de Protection des Personnes (CPP) prior to undertaking this study [1]. The Editorial Board determined that approval from the CPP is a mandatory national requirement and that the approval received from the Ethics Committee of IHU Méditerranée Infection was insufficient in this situation. As a result, the Editorial Office and Editorial Board have decided to retract this article as per MDPI’s retraction policy (https://www.mdpi.com/ethics#_bookmark30) and in line with the Committee on Publication Ethics retraction guidelines (https://publicationethics.org/retraction-guidelines). 

This retraction was approved by the Editor-in-Chief of the *Journal of Clinical Medicine*. 

The authors did not agree to this retraction. 
